# Crystal structure of a seven-coordinate manganese(II) complex with tris­(pyridin-2-ylmeth­yl)amine (TMPA)

**DOI:** 10.1107/S2056989018009611

**Published:** 2018-07-10

**Authors:** Steven T. Frey, Hillary A. Ramirez, Manpreet Kaur, Jerry P. Jasinski

**Affiliations:** a Department of Chemistry, Skidmore College, 815 North Broadway, Saratoga Springs, NY 12866, USA; b Department of Chemistry, Keene State College, 229 Main Street, Keene, NH 03435-2001, USA

**Keywords:** crystal structure, manganese(II), tripodal ligand, seven-coordinate

## Abstract

Structural analysis of (acetato-κ^2^ *O*,*O*′)(methanol-κ*O*)[tris­(pyridin-2-ylmeth­yl)amine-κ^4^ *N*,*N*′,*N*′′,*N*′′′]manganese(II) tetraphenyl­borate, [Mn(C_2_H_3_O_2_)(C_18_H_18_N_4_)(CH_3_OH)](C_24_H_20_B) or [Mn(TMPA)(Ac)(CH_3_OH)]BPh_4_ [TMPA = tris­(pyridin-2-ylmeth­yl)amine, Ac = acetate, BPh_4_ = tetra­phenyl­borate] by single-crystal X-ray diffraction reveals a complex cation with tetra­dentate coordination of the tripodal TMPA ligand, bidentate coordination of the Ac ligand and monodentate coordination of the methanol ligand to a single Mn^II^ center, balanced in charge by the presence of a tetra­phenyl­borate anion. The Mn^II^ complex has a distorted penta­gonal–bipyramidal geometry, in which the central amine nitro­gen and two pyridyl N atoms of the TMPA ligand, and two oxygen atoms of the acetate ligand occupy positions in the penta­gonal plane, while the third pyridyl nitro­gen of TMPA and the oxygen from the methanol ligand occupy the axial positions. Within the complex, the acetate O atoms participate in weak C—H⋯O hydrogen-bonding inter­actions with neighboring pyridyl moieties. In the crystal, complexes form dimers by pairs of O—H⋯O hydrogen bonds between the coordinated methanol of one complex and an acetate oxygen of the other, and weak π-stacking inter­actions between pyridine rings. Separate dimers then undergo additional π-stacking inter­actions between the pyridine rings of one moiety and either the pyridine or phenyl rings of another moiety that further stabilize the crystal.

## Abstract 2

The crystal structure of [Mn(TMPA)(Ac)(CH_3_OH)]BPh_4_ (TMPA = tris­(pyridin-2-yl­meth­yl)amine, Ac = acetate, BPh_4_ = tetra­phenyl­borate) has been determined. The structure reveals a seven-coordinate Mn^II^ center with distorted, penta­gonal bipyramidal geometry.

## Chemical context   

A variety of manganese(II/III) complexes have been studied as structural and functional mimics of superoxide dismutase (SOD) enzymes (Batinić-Haberle *et al.*, 2010 ▸, 2014 ▸; Iranzo, 2011 ▸; Bani & Bencini, 2012 ▸; Miriyala *et al.*, 2012 ▸; Policar, 2016 ▸). The efficacy of these mimics is reliant on their stability in aqueous solution, retention of open or substitutional coordination sites on the manganese ion, and Mn^III^/Mn^II^ redox potential lying in the narrow range of 0.2–0.4 V *versus* a normal hydrogen electrode (Iranzo, 2011 ▸; Policar, 2016 ▸). These factors are directly related to the nature of the ligands employed, their coordinating atoms, and the geometry of the coordination sphere (Policar, 2016 ▸).

One family of manganese(II) complexes that has been studied incorporates N-centered, tripodal, tetra­dentate ligands (Policar *et al.*, 2001 ▸; Durot *et al.*, 2005 ▸; Ribeiro *et al.*, 2015 ▸). These ligands can be readily synthesized to provide a variety of N and O donors that give rise to the structural diversity of their metal complexes (Policar *et al.*, 2001 ▸). With that in mind, we have begun to examine manganese(II) complexes with tripodal ligands containing either pyridine or quinoline groups. Herein, we report the synthesis and structural characterization of [Mn(TMPA)(Ac)(CH_3_OH)]BPh_4_ [TMPA = tris­(pyridin-2-yl­meth­yl)amine, Ac = acetate, BPh_4_ = tetra­phenyl­borate]. This compound is prepared by a two-step process (see reaction scheme) in which manganese(II) acetate is reacted with TMPA in a methanol solution, followed by anion exchange with sodium tetra­phenyl­borate. The resulting monomeric complex exhibits notable characteristics including a high coordination number of seven, a distorted penta­gonal–bipyramidyl geometry, asymmetric bidentate coordination of the acetate ligand, and coordination by a methanol ligand.
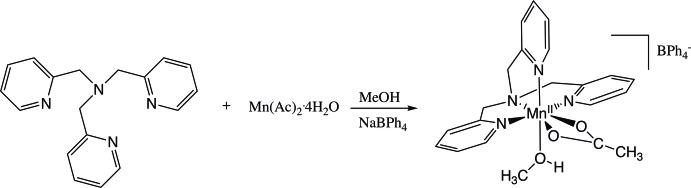


## Structural commentary   

**Figure 1:**
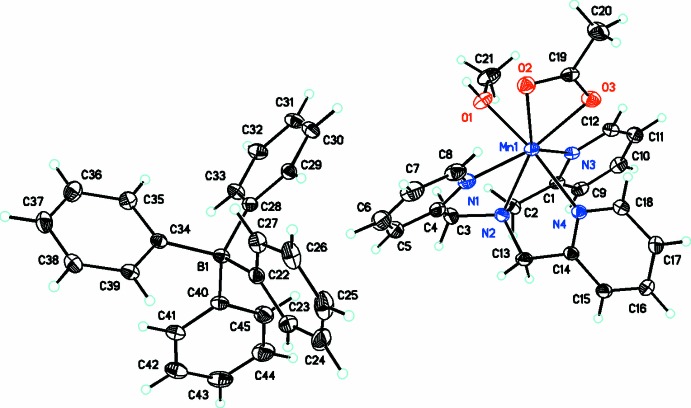
Mol­ecular structure of [Mn(TMPA)(Ac)(CH_3_OH)]BPh_4_ [TMPA = tris(pyridin-2-yl­meth­yl)amine, Ac = acetate, BPh_4_ = tetra­phenyl­borate] with atom labels. Displacement ellipsoids are drawn at the 30% probability level.

The title compound (Fig. 1 ▸), which consists of the [Mn(TMPA)(Ac)(CH_3_OH)]^+^ monocation and tetra­phenyl­borate counter-anion, crystallizes in the triclinic space group *P* 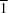. The manganese(II) ion is hepta­coordinate with a geometry that is best described as a distorted penta­gonal bipyramid. While this is a high coordination number for a first row transition metal ion, seven-coordinate manganese(II) complexes with N-donor ligands have been described previously (Deroche *et al.*., 1996 ▸; Policar *et al.*, 2001 ▸; Lessa *et al.*, 2007 ▸; Dees *et al.*, 2007 ▸; Wu *et al.*, 2010 ▸; Lieb *et al.*, 2013 ▸). The TMPA ligand is tetra­dentate, with its central N2 and two pyridyl nitro­gen atoms (N1 and N3) in the penta­gonal plane, and the third pyridyl nitro­gen (N4) occupying an axial position. The remaining two positions in the penta­gonal plane are completed by the bidentate coordination of the acetate ligand (O2 and O3), while the final axial position is occupied by O1 of the methanol ligand. Distortion of the penta­gonal–bipyramidal geometry of the coordination sphere is produced by the bite angles of the TMPA and acetate chelate rings. For example, the N2—Mn1—N4 bond angle [75.20 (4)°] of the five-membered metallacycle spanning an equatorial and axial position, is significantly reduced from 90° (Table 1 ▸). This results in a *trans* O1—Mn1—N4 angle of 166.95 (5)°. Likewise, the O2—Mn1—O3 bond angle [54.74 (4)°] that results from bidentate coordination of the acetate ligand is significantly reduced from the ideal 72° bond angle within the penta­gonal plane. The O2—Mn1—O3 plane is also twisted outside of the the penta­gonal plane by approximately 10° as a result of weak intra­molecular C—H⋯O hydrogen-bonding inter­actions with neighboring pyridyl rings (Table 2 ▸). What is perhaps most remarkable about the bidentate coordination of the acetate ligand is how asymmetric it is. The Mn1—O2 and Mn1—O3 bond lengths differ from each other by 0.3005 Å. This does not appear to result from steric hindrance, but may be due to an inter­molecular hydrogen-bonding inter­action between the O2 acetate oxygen of one complex and the hydroxyl hydrogen of the coordinated methanol of another, having the effect of lengthening the Mn1—O2 bond. The bond between the manganese(II) ion and the central TMPA nitro­gen, Mn1—N2 is also considerably long at 2.4092 (13) Å. This elongation has been observed in other manganese(II) complexes with tripodal, tetra­dentate ligands (Deroche *et al.*, 1996 ▸; Wu *et al.*, 2010 ▸). The other Mn—O and Mn—N bonds fall into the range 2.2–2.3 Å, which is typical of manganese(II) complexes (Deroche *et al.*, 1996 ▸; Policar *et al.*, 2001 ▸; Lessa *et al.*, 2007 ▸; Dees *et al.*, 2007 ▸; Wu *et al.*, 2010 ▸; Lieb *et al.*, 2012 ▸).

## Supra­molecular features   

**Figure 2:**
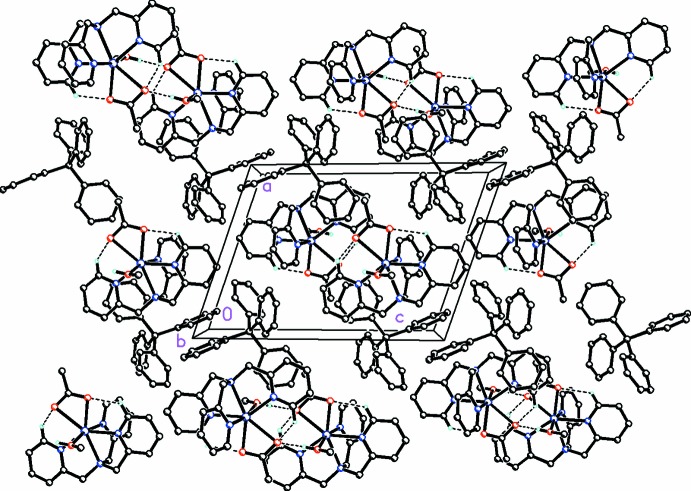
A view along the *b* axis of the crystal packing of the title compound. The intra­molecular O—H⋯O and inter­molecular C—H⋯O hydrogen bonds (Table 2 ▸) are shown as dashed lines.

Within the crystal, dimerization of complexes occurs by the formation of a pair of inter­moleclular O—H⋯O hydrogen bonds (Table 2 ▸) between the coordinated methanol of one complex and an acetate oxygen of another (Fig. 2 ▸) forming an 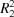
(12) ring-motif inter­action. Within a dimer, weak π-stacking inter­actions between pyridine rings (*Cg*2⋯*Cg*3) can be detected. Separate dimers then undergo additional π-stacking between the pyridine rings of one moiety and the phenyl rings of a second (*Cg*1⋯*Cg*7 and *Cg*3⋯*Cg*4) as well as between the pyridine rings of separate moieties (*Cg*4⋯*Cg*6) [where *Cg*1, *Cg*2, *Cg*3, *Cg*4, *Cg*6, and *Cg*7 are the centroids of the N1/C4–C8, N3/C1/C9/C10–C12, N4/C14–C18, C22–C27, C34–C39, and C40–C45 rings, respectively] that further stabilize the crystal packing. In addition, weak slipped parallel C—H⋯π [C2—H2*B*⋯*Cg*6, *X*—H, π = 62°; C38—H38⋯*Cg*4, *X*—H, π = 61°; C42—H42⋯*Cg*3, *X*—H, π = 38°] (Table 2 ▸) inter­molecular inter­actions are also present and contibute additionally to the crystal packing.

## Database survey   

A search of the Cambridge Structural Database (Version 5.39; last update May 2018; Groom *et al.* 2016 ▸) for manganese(II) complexes containing TMPA revealed 17 structures related to the title compound. Twelve of these are dimeric in nature and contain a variety of bridging ligands (Oshio *et al.*, 1993 ▸; Xiang *et al.*, 1998 ▸; Shin *et al.*, 2010 ▸; Barros *et al.*, 2013 ▸; Khullar & Mandal, 2013 ▸), including one with bridging acetate ligands (Oshio *et al.*, 1993 ▸). The remaining five structures are monomeric and include monodentate ligands in addition to TMPA (Oshio *et al.*, 1993 ▸; Hitomi *et al.*, 2005 ▸; Duboc *et al.*, 2008 ▸; Shin *et al.*, 2010 ▸; Ogo *et al.*, 2014 ▸). Of the 17 structures, 16 are six-coordinate with respect to the manganese(II) centers, while the remaining structure has a five-coordinate manganese(II) center. None of these structures reveal coordination numbers greater than six. However, a separate literature search identified an eight-coordinate complex in which one mangan­ese(II) ion is coordinated to two tetra­dentate TMPA ligands (Gultneh *et al.*, 1993 ▸).

## Synthesis and crystallization   

All chemicals were obtained from commercial sources and used without further preparation. The water used was deionized. The ^1^H NMR spectrum was recorded with a JEOL ECX-300 NMR spectrometer and referenced against the ^1^H peak of the chloro­form solvent. IR spectra were recorded with a Perkin Elmer Spectrum 100 FT–IR.

**Tris(pyridin-2-yl­meth­yl)amine (TMPA)**. In a 250 mL round-bottom flask, 10 g (61 mmol) picolyl chloride hydro­chloride was dissolved in 20 mL H_2_O and cooled to 273 K in an ice bath. A solution of 5.0 g (120 mmol) NaOH in 20 mL H_2_O was added dropwise under stirring. Following this, a solution of 2-methyl­amino­pyridine (3.3 g, 31 mmol) in CH_2_Cl_2_ (40 mL) was added. The reaction mixture was then removed from the ice bath, capped, and allowed to stir vigorously for five days. The CH_2_Cl_2_ layer was then separated, washed twice with brine, and dried over anhydrous sodium sulfate. The solution was filtered and concentrated on a rotary evaporator producing 6.5 g of a red–brown oil that solidified upon cooling. The crude product was chromatographed on alumina (chromatographic grade, 80–200 mesh) eluting with 20:1 ethyl acetate/methanol, producing 4.9 g (55%) of a pure, golden oil that solidified upon standing. ^1^H NMR (CDCl_3_, 300 MHz) δ 3.88 (*s*, 6H), 7.15 (*t*, 3H), 7.57–7.69 (*m*, 6H), 8.53 (*d*, 3H).

**[Mn(TMPA)(Ac)(CH_3_OH)]BPh_4_**. In a 100 mL round-bottom flask, 0.41 g (1.4 mmol) TMPA was dissolved in 10 mL of methanol. To this solution, 0.35 g (1.4 mmol) of mangan­ese(II) acetate tetra­hydrate was added, and the solution was brought to reflux for 20 minutes. A solution of 0.48 g (1.4 mmol) of sodium tetra­phenyl­borate in 10 mL of methanol was then added dropwise to the warm reaction mixture. A precipitate formed during this addition. The reaction mixture was cooled to room temperature and filtered to produce tan microcrystals that were washed twice with cold methanol and air dried to give 0.75 g (74%) of product. The filtrate was then capped and placed in the refrigerator to promote further crystallization. After several days, crystals suitable for X-ray diffraction formed, which gave an IR spectrum identical to the original product. IR (ATR, cm^−1^) 3000–3053 (aromatic C—H, *w*), 1589 (C—O, *s*), 1425 (C—O, *s*), 731 (BPh_4_^−^, *s*), 701 (BPh_4_^−^, *s*).

## Refinement   

Crystal data, data collection and structure refinement details are summarized in Table 3 ▸. The hy­droxy H atom was located in a difference-Fourier map and refined with the distance restraint O1—H1 = 0.85 ± 0.01 and with *U*_iso_(H) = 1.2*U*_eq_(O). C-bound H atoms were positioned geometrically and refined as riding: C—H = 0.95–0.99 Å with *U*_iso_(H) = 1.2*U*_eq_(C) or 1.5*U*_eq_(C-meth­yl).

## Supplementary Material

Crystal structure: contains datablock(s) I. DOI: 10.1107/S2056989018009611/tx2006sup1.cif

Structure factors: contains datablock(s) I. DOI: 10.1107/S2056989018009611/tx2006Isup2.hkl

CCDC reference: 1853486

Additional supporting information:  crystallographic information; 3D view; checkCIF report

## Figures and Tables

**Table 1:** Selected geometric parameters (Å, °)

Mn1—O1	2.1941 (12)	Mn1—N2	2.4092 (13)
Mn1—O2	2.5009 (12)	Mn1—N3	2.3022 (13)
Mn1—O3	2.2004 (13)	Mn1—N4	2.2496 (13)
Mn1—N1	2.2769 (15)		
			
O1—Mn1—O2	81.52 (4)	O2—Mn1—O3	54.74 (4)
O1—Mn1—N4	166.95 (5)	N2—Mn1—N4	75.20 (4)

**Table 2:** Hydrogen-bond geometry and π–π stacking interactions (Å, °) *Cg*1, *Cg*2, *Cg*3, *Cg*4, *Cg*6, and *Cg*7 are the centroids of the N1/C4–C8, N3/C1/C9/C10–C12, N4/C14–C18, C22–C27, C34–C39, and C40–C45 rings, respectively.

*D*—H⋯*A*	*D*—H	H⋯*A*	*D*⋯*A*	*D*—H⋯*A*
O1—H1⋯O2^i^	0.86 (1)	1.79 (1)	2.6480 (17)	176 (2)
C8—H8⋯O2	0.95	2.45	3.056 (2)	121
C12—H12⋯O3	0.95	2.35	2.987 (2)	124
C2—H2*B*⋯*Cg*6^ii^	0.99	2.70	3.6260 (18)	156
C38—H38⋯*Cg*4^iii^	0.95	2.81	3.7135 (19)	158
C42—H42⋯*Cg*3^iv^	0.95	2.96	3.659 (2)	131
*Cg*1⋯*Cg*7^iv^			4.2073 (11)	
*Cg*2⋯*Cg*3			4.6125 (10)	
*Cg*3⋯*Cg*4^v^			4.2267 (12)	
*Cg*4⋯*Cg*6^iii^			5.0645 (11)	

Symmetry codes: (i) 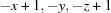; (ii) 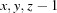; (iii) 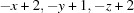; (iv) 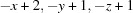; (v) 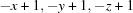.

**Table 3:** Experimental details

Crystal data	
Chemical formula	[Mn(C_2_H_3_O_2_)(C_18_H_18_N_4_)(CH_4_O)](C_24_H_20_B)
*M*_r_	755.60
Crystal system, space group	Triclinic, *P* 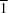
Temperature (K)	173
*a*, *b*, *c* (Å)	11.3885 (8), 11.7598 (7), 15.6703 (10)
α, β, γ (°)	82.041 (5), 70.671 (6), 85.870 (5)
*V* (Å^3^)	1960.5 (2)
*Z*	2
Radiation type	Mo *K*α
μ (mm^−1^)	0.38
Crystal size (mm)	0.44 × 0.38 × 0.26

Data collection	
Diffractometer	Rigaku Oxford Diffraction
Absorption correction	Multi-scan (*CrysAlis PRO*; Agilent, 2014 ▸)
*T*_min_, *T*_max_	0.836, 1.000
No. of measured, independent and observed [*I* > 2σ(*I*)] reflections	24707, 12901, 9324
*R*_int_	0.029
(sin θ/λ)_max_ (Å^−1^)	0.763

Refinement	
*R*[*F* ^2^ > 2σ(*F* ^2^)], *wR*(*F* ^2^), *S*	0.047, 0.122, 1.03
No. of reflections	12901
No. of parameters	492
No. of restraints	3
H-atom treatment	H-atom parameters constrained
Δρ_max_, Δρ_min_ (e Å^−3^)	0.36, −0.30

Computer programs: *CrysAlis PRO* (Agilent, 2014 ▸), *SHELXT* (Sheldrick, 2015*a* ▸), *SHELXL2014* (Sheldrick, 2015*b* ▸) and *OLEX2* (Dolomanov *et al.*, 2009 ▸).

## supplementary crystallographic information

### Crystal data

**Table:**

[Mn(C_2_H_3_O_2_)(C_18_H_18_N_4_)(CH_4_O)](C_24_H_20_B)	*Z* = 2
*M**_r_* = 755.60	*F*(000) = 794
Triclinic, *P*1	*D*_x_ = 1.280 Mg m^−^^3^
*a* = 11.3885 (8) Å	Mo *K*α radiation, λ = 0.71073 Å
*b* = 11.7598 (7) Å	Cell parameters from 6236 reflections
*c* = 15.6703 (10) Å	θ = 3.5–32.2°
α = 82.041 (5)°	µ = 0.38 mm^−^^1^
β = 70.671 (6)°	*T* = 173 K
γ = 85.870 (5)°	Prism, orange
*V* = 1960.5 (2) Å^3^	0.44 × 0.38 × 0.26 mm

### Data collection

**Table:**

Rigaku Oxford Diffraction diffractometer	12901 independent reflections
Radiation source: Enhance (Mo) X-ray Source	9324 reflections with *I* > 2σ(*I*)
Graphite monochromator	*R*_int_ = 0.029
Detector resolution: 16.0416 pixels mm^-1^	θ_max_ = 32.8°, θ_min_ = 3.1°
ω scans	*h* = −16→16
Absorption correction: multi-scan (CrysAlis PRO; Agilent, 2014)	*k* = −17→17
*T*_min_ = 0.836, *T*_max_ = 1.000	*l* = −23→23
24707 measured reflections	

### Refinement

**Table:**

Refinement on *F*^2^	Primary atom site location: structure-invariant direct methods
Least-squares matrix: full	Hydrogen site location: inferred from neighbouring sites
*R*[*F*^2^ > 2σ(*F*^2^)] = 0.047	H-atom parameters constrained
*wR*(*F*^2^) = 0.122	*w* = 1/[σ^2^(*F*_o_^2^) + (0.0476*P*)^2^ + 0.5222*P*] where *P* = (*F*_o_^2^ + 2*F*_c_^2^)/3
*S* = 1.03	(Δ/σ)_max_ = 0.004
12901 reflections	Δρ_max_ = 0.36 e Å^−^^3^
492 parameters	Δρ_min_ = −0.30 e Å^−^^3^
3 restraints	

### Special details

**Table:**

Geometry. All esds (except the esd in the dihedral angle between two l.s. planes) are estimated using the full covariance matrix. The cell esds are taken into account individually in the estimation of esds in distances, angles and torsion angles; correlations between esds in cell parameters are only used when they are defined by crystal symmetry. An approximate (isotropic) treatment of cell esds is used for estimating esds involving l.s. planes.

### Fractional atomic coordinates and isotropic or equivalent isotropic displacement parameters (Å^2^)

**Table:**

	*x*	*y*	*z*	*U*_iso_*/*U*_eq_	
Mn1	0.56866 (2)	0.16825 (2)	0.32745 (2)	0.02659 (7)	
O1	0.63017 (13)	−0.01175 (10)	0.34698 (8)	0.0391 (3)	
H1	0.613 (2)	−0.0420 (12)	0.4030 (7)	0.059*	
O2	0.41990 (12)	0.11321 (10)	0.48365 (8)	0.0385 (3)	
O3	0.36694 (11)	0.14978 (11)	0.36100 (8)	0.0388 (3)	
N1	0.68813 (14)	0.21241 (12)	0.40889 (10)	0.0360 (3)	
N2	0.77041 (12)	0.22294 (11)	0.22297 (9)	0.0287 (3)	
N3	0.57535 (12)	0.13981 (10)	0.18346 (8)	0.0267 (3)	
N4	0.54497 (12)	0.35519 (11)	0.27955 (8)	0.0263 (3)	
C1	0.68402 (14)	0.15094 (12)	0.11539 (10)	0.0270 (3)	
C2	0.79743 (15)	0.16420 (15)	0.14101 (11)	0.0344 (3)	
H2A	0.8349	0.0873	0.1520	0.041*	
H2B	0.8593	0.2084	0.0894	0.041*	
C3	0.85963 (16)	0.18541 (15)	0.27130 (12)	0.0367 (4)	
H3A	0.9397	0.2234	0.2387	0.044*	
H3B	0.8749	0.1014	0.2717	0.044*	
C4	0.81202 (17)	0.21393 (14)	0.36774 (12)	0.0365 (4)	
C5	0.8922 (2)	0.23550 (16)	0.41321 (15)	0.0491 (5)	
H5	0.9795	0.2366	0.3829	0.059*	
C6	0.8438 (3)	0.25523 (18)	0.50269 (16)	0.0596 (6)	
H6	0.8973	0.2682	0.5355	0.071*	
C7	0.7165 (3)	0.25604 (18)	0.54439 (15)	0.0568 (6)	
H7	0.6811	0.2711	0.6059	0.068*	
C8	0.6414 (2)	0.23465 (16)	0.49564 (12)	0.0444 (4)	
H8	0.5537	0.2357	0.5243	0.053*	
C9	0.69333 (16)	0.14408 (13)	0.02599 (10)	0.0325 (3)	
H9	0.7714	0.1523	−0.0211	0.039*	
C10	0.58756 (18)	0.12519 (14)	0.00635 (11)	0.0370 (4)	
H10	0.5917	0.1203	−0.0546	0.044*	
C11	0.47552 (17)	0.11350 (14)	0.07615 (12)	0.0356 (4)	
H11	0.4014	0.0999	0.0644	0.043*	
C12	0.47377 (15)	0.12198 (13)	0.16317 (11)	0.0304 (3)	
H12	0.3965	0.1148	0.2112	0.037*	
C13	0.76954 (15)	0.34957 (14)	0.20102 (12)	0.0343 (3)	
H13A	0.8208	0.3691	0.1365	0.041*	
H13B	0.8098	0.3824	0.2389	0.041*	
C14	0.64285 (14)	0.40629 (13)	0.21531 (10)	0.0265 (3)	
C15	0.63108 (16)	0.51242 (14)	0.16752 (11)	0.0350 (4)	
H15	0.7008	0.5458	0.1207	0.042*	
C16	0.51625 (18)	0.56882 (15)	0.18919 (14)	0.0424 (4)	
H16	0.5064	0.6423	0.1580	0.051*	
C17	0.41608 (17)	0.51760 (15)	0.25640 (13)	0.0389 (4)	
H17	0.3367	0.5555	0.2731	0.047*	
C18	0.43378 (15)	0.41047 (13)	0.29869 (11)	0.0304 (3)	
H18	0.3642	0.3739	0.3435	0.036*	
C19	0.33901 (15)	0.11975 (13)	0.44512 (10)	0.0298 (3)	
C20	0.20607 (17)	0.09291 (19)	0.49920 (13)	0.0473 (5)	
H20A	0.1625	0.1621	0.5240	0.071*	
H20B	0.2037	0.0322	0.5493	0.071*	
H20C	0.1652	0.0669	0.4596	0.071*	
C21	0.6314 (2)	−0.10245 (16)	0.29597 (14)	0.0516 (5)	
H21A	0.6816	−0.0813	0.2322	0.077*	
H21B	0.5461	−0.1167	0.2996	0.077*	
H21C	0.6675	−0.1721	0.3208	0.077*	
C22	0.87749 (14)	0.44057 (12)	0.82076 (11)	0.0275 (3)	
C23	0.88441 (18)	0.54783 (14)	0.76863 (13)	0.0385 (4)	
H23	0.9513	0.5613	0.7131	0.046*	
C24	0.7964 (2)	0.63588 (16)	0.79538 (17)	0.0520 (5)	
H24	0.8044	0.7079	0.7584	0.062*	
C25	0.6982 (2)	0.61887 (17)	0.87495 (16)	0.0518 (5)	
H25	0.6373	0.6783	0.8924	0.062*	
C26	0.68858 (18)	0.51532 (17)	0.92930 (14)	0.0433 (4)	
H26	0.6213	0.5030	0.9847	0.052*	
C27	0.77791 (15)	0.42884 (14)	0.90271 (11)	0.0328 (3)	
H27	0.7712	0.3586	0.9419	0.039*	
C28	0.90673 (13)	0.22992 (12)	0.76412 (9)	0.0239 (3)	
C29	0.77827 (14)	0.22214 (13)	0.78503 (10)	0.0260 (3)	
H29	0.7251	0.2787	0.8177	0.031*	
C30	0.72484 (15)	0.13489 (15)	0.75994 (11)	0.0323 (3)	
H30	0.6369	0.1328	0.7760	0.039*	
C31	0.79897 (16)	0.05108 (15)	0.71164 (11)	0.0343 (4)	
H31	0.7629	−0.0084	0.6942	0.041*	
C32	0.92625 (16)	0.05606 (14)	0.68956 (11)	0.0334 (3)	
H32	0.9787	−0.0005	0.6564	0.040*	
C33	0.97842 (15)	0.14322 (13)	0.71539 (11)	0.0299 (3)	
H33	1.0664	0.1442	0.6994	0.036*	
C34	1.02371 (13)	0.28206 (12)	0.87796 (10)	0.0238 (3)	
C35	1.05266 (15)	0.16588 (13)	0.89976 (11)	0.0305 (3)	
H35	1.0374	0.1108	0.8660	0.037*	
C36	1.10278 (16)	0.12836 (15)	0.96888 (12)	0.0370 (4)	
H36	1.1217	0.0490	0.9809	0.044*	
C37	1.12527 (15)	0.20534 (16)	1.02014 (12)	0.0361 (4)	
H37	1.1606	0.1798	1.0668	0.043*	
C38	1.09545 (15)	0.32066 (15)	1.00249 (11)	0.0327 (3)	
H38	1.1088	0.3747	1.0379	0.039*	
C39	1.04616 (14)	0.35686 (13)	0.93316 (11)	0.0286 (3)	
H39	1.0265	0.4363	0.9223	0.034*	
C40	1.09510 (15)	0.37221 (12)	0.70190 (10)	0.0277 (3)	
C41	1.21400 (15)	0.38527 (13)	0.70467 (11)	0.0314 (3)	
H41	1.2281	0.3688	0.7616	0.038*	
C42	1.31390 (17)	0.42153 (16)	0.62752 (13)	0.0414 (4)	
H42	1.3934	0.4299	0.6329	0.050*	
C43	1.2972 (2)	0.44500 (17)	0.54396 (13)	0.0484 (5)	
H43	1.3649	0.4689	0.4912	0.058*	
C44	1.1809 (2)	0.43339 (18)	0.53774 (13)	0.0509 (5)	
H44	1.1680	0.4498	0.4804	0.061*	
C45	1.08230 (18)	0.39766 (16)	0.61519 (12)	0.0403 (4)	
H45	1.0030	0.3902	0.6091	0.048*	
B1	0.97542 (15)	0.33157 (13)	0.79157 (11)	0.0245 (3)	

### Atomic displacement parameters (Å^2^)

**Table:**

	*U*^11^	*U*^22^	*U*^33^	*U*^12^	*U*^13^	*U*^23^
Mn1	0.02860 (12)	0.02566 (12)	0.02341 (11)	−0.00437 (8)	−0.00563 (9)	−0.00115 (8)
O1	0.0554 (8)	0.0279 (6)	0.0311 (6)	0.0023 (5)	−0.0115 (6)	−0.0019 (5)
O2	0.0386 (7)	0.0386 (7)	0.0363 (6)	−0.0014 (5)	−0.0102 (5)	−0.0024 (5)
O3	0.0376 (7)	0.0460 (7)	0.0263 (6)	−0.0104 (5)	−0.0018 (5)	0.0002 (5)
N1	0.0463 (9)	0.0316 (7)	0.0326 (7)	−0.0079 (6)	−0.0160 (6)	−0.0003 (5)
N2	0.0265 (6)	0.0273 (6)	0.0319 (7)	−0.0024 (5)	−0.0086 (5)	−0.0034 (5)
N3	0.0292 (6)	0.0236 (6)	0.0241 (6)	−0.0041 (5)	−0.0042 (5)	−0.0019 (5)
N4	0.0280 (6)	0.0262 (6)	0.0251 (6)	−0.0031 (5)	−0.0085 (5)	−0.0036 (5)
C1	0.0289 (7)	0.0203 (6)	0.0270 (7)	−0.0010 (5)	−0.0030 (6)	−0.0016 (5)
C2	0.0253 (8)	0.0398 (9)	0.0348 (8)	−0.0005 (6)	−0.0030 (6)	−0.0107 (7)
C3	0.0305 (8)	0.0372 (9)	0.0446 (10)	−0.0005 (7)	−0.0156 (7)	−0.0037 (7)
C4	0.0451 (10)	0.0272 (8)	0.0427 (9)	−0.0071 (7)	−0.0231 (8)	0.0026 (6)
C5	0.0594 (13)	0.0370 (10)	0.0644 (13)	−0.0122 (8)	−0.0396 (11)	0.0031 (9)
C6	0.0905 (19)	0.0443 (11)	0.0657 (15)	−0.0172 (11)	−0.0548 (14)	0.0016 (10)
C7	0.0952 (19)	0.0447 (11)	0.0406 (11)	−0.0183 (11)	−0.0340 (12)	−0.0010 (8)
C8	0.0651 (13)	0.0388 (9)	0.0310 (9)	−0.0133 (8)	−0.0169 (9)	0.0000 (7)
C9	0.0385 (9)	0.0268 (7)	0.0245 (7)	−0.0002 (6)	−0.0002 (6)	−0.0030 (6)
C10	0.0500 (11)	0.0342 (8)	0.0276 (8)	−0.0006 (7)	−0.0125 (7)	−0.0070 (6)
C11	0.0402 (9)	0.0337 (8)	0.0373 (9)	−0.0029 (7)	−0.0160 (7)	−0.0092 (7)
C12	0.0299 (8)	0.0296 (7)	0.0296 (7)	−0.0064 (6)	−0.0054 (6)	−0.0042 (6)
C13	0.0279 (8)	0.0296 (8)	0.0400 (9)	−0.0061 (6)	−0.0049 (7)	0.0011 (6)
C14	0.0288 (7)	0.0273 (7)	0.0247 (7)	−0.0056 (6)	−0.0092 (6)	−0.0034 (5)
C15	0.0370 (9)	0.0350 (8)	0.0344 (8)	−0.0118 (7)	−0.0154 (7)	0.0057 (6)
C16	0.0436 (10)	0.0337 (9)	0.0539 (11)	−0.0046 (7)	−0.0266 (9)	0.0104 (8)
C17	0.0343 (9)	0.0360 (9)	0.0493 (10)	0.0015 (7)	−0.0194 (8)	−0.0017 (7)
C18	0.0288 (8)	0.0315 (8)	0.0306 (8)	−0.0032 (6)	−0.0086 (6)	−0.0043 (6)
C19	0.0329 (8)	0.0229 (7)	0.0279 (7)	−0.0030 (6)	−0.0017 (6)	−0.0038 (5)
C20	0.0340 (9)	0.0643 (13)	0.0331 (9)	−0.0098 (8)	0.0030 (7)	−0.0010 (8)
C21	0.0766 (15)	0.0312 (9)	0.0400 (10)	−0.0003 (9)	−0.0088 (10)	−0.0067 (7)
C22	0.0298 (8)	0.0228 (7)	0.0353 (8)	−0.0003 (5)	−0.0167 (6)	−0.0068 (6)
C23	0.0437 (10)	0.0255 (8)	0.0508 (10)	−0.0020 (7)	−0.0224 (8)	−0.0015 (7)
C24	0.0655 (14)	0.0262 (9)	0.0791 (15)	0.0092 (8)	−0.0450 (13)	−0.0071 (9)
C25	0.0553 (13)	0.0427 (11)	0.0744 (15)	0.0252 (9)	−0.0403 (12)	−0.0301 (10)
C26	0.0363 (9)	0.0539 (11)	0.0498 (11)	0.0155 (8)	−0.0217 (8)	−0.0294 (9)
C27	0.0329 (8)	0.0333 (8)	0.0356 (8)	0.0059 (6)	−0.0139 (7)	−0.0121 (6)
C28	0.0258 (7)	0.0229 (6)	0.0228 (6)	−0.0036 (5)	−0.0074 (5)	−0.0022 (5)
C29	0.0254 (7)	0.0287 (7)	0.0241 (7)	−0.0019 (5)	−0.0082 (5)	−0.0032 (5)
C30	0.0270 (8)	0.0430 (9)	0.0277 (7)	−0.0105 (6)	−0.0078 (6)	−0.0048 (6)
C31	0.0387 (9)	0.0379 (9)	0.0272 (7)	−0.0170 (7)	−0.0077 (6)	−0.0061 (6)
C32	0.0371 (9)	0.0296 (8)	0.0327 (8)	−0.0054 (6)	−0.0066 (7)	−0.0112 (6)
C33	0.0261 (7)	0.0310 (8)	0.0336 (8)	−0.0037 (6)	−0.0081 (6)	−0.0095 (6)
C34	0.0187 (6)	0.0241 (6)	0.0263 (7)	−0.0019 (5)	−0.0035 (5)	−0.0043 (5)
C35	0.0314 (8)	0.0282 (7)	0.0323 (8)	0.0028 (6)	−0.0098 (6)	−0.0084 (6)
C36	0.0355 (9)	0.0330 (8)	0.0415 (9)	0.0100 (7)	−0.0134 (7)	−0.0047 (7)
C37	0.0270 (8)	0.0482 (10)	0.0347 (8)	0.0034 (7)	−0.0130 (7)	−0.0046 (7)
C38	0.0282 (8)	0.0393 (9)	0.0331 (8)	−0.0051 (6)	−0.0109 (6)	−0.0080 (7)
C39	0.0268 (7)	0.0258 (7)	0.0334 (8)	−0.0046 (5)	−0.0087 (6)	−0.0053 (6)
C40	0.0312 (8)	0.0214 (7)	0.0298 (7)	−0.0058 (5)	−0.0072 (6)	−0.0047 (5)
C41	0.0291 (8)	0.0293 (7)	0.0326 (8)	−0.0060 (6)	−0.0035 (6)	−0.0072 (6)
C42	0.0326 (9)	0.0410 (9)	0.0434 (10)	−0.0115 (7)	0.0009 (7)	−0.0089 (8)
C43	0.0513 (12)	0.0440 (10)	0.0369 (10)	−0.0175 (8)	0.0060 (8)	−0.0043 (8)
C44	0.0677 (14)	0.0531 (12)	0.0283 (9)	−0.0187 (10)	−0.0109 (9)	0.0040 (8)
C45	0.0447 (10)	0.0445 (10)	0.0324 (9)	−0.0129 (8)	−0.0128 (7)	−0.0008 (7)
B1	0.0241 (8)	0.0207 (7)	0.0282 (8)	−0.0029 (6)	−0.0069 (6)	−0.0041 (6)

### Geometric parameters (Å, º)

**Table:**

Mn1—O1	2.1941 (12)	C20—H20A	0.9800
Mn1—O2	2.5009 (12)	C20—H20B	0.9800
Mn1—O3	2.2004 (13)	C20—H20C	0.9800
Mn1—N1	2.2769 (15)	C21—H21A	0.9800
Mn1—N2	2.4092 (13)	C21—H21B	0.9800
Mn1—N3	2.3022 (13)	C21—H21C	0.9800
Mn1—N4	2.2496 (13)	C22—C23	1.397 (2)
O1—H1	0.863 (9)	C22—C27	1.401 (2)
O1—C21	1.415 (2)	C22—B1	1.648 (2)
O2—C19	1.251 (2)	C23—H23	0.9500
O3—C19	1.2541 (19)	C23—C24	1.395 (3)
N1—C4	1.343 (2)	C24—H24	0.9500
N1—C8	1.341 (2)	C24—C25	1.373 (3)
N2—C2	1.475 (2)	C25—H25	0.9500
N2—C3	1.467 (2)	C25—C26	1.375 (3)
N2—C13	1.481 (2)	C26—H26	0.9500
N3—C1	1.3406 (19)	C26—C27	1.389 (2)
N3—C12	1.334 (2)	C27—H27	0.9500
N4—C14	1.3428 (19)	C28—C29	1.396 (2)
N4—C18	1.342 (2)	C28—C33	1.404 (2)
C1—C2	1.498 (2)	C28—B1	1.651 (2)
C1—C9	1.383 (2)	C29—H29	0.9500
C2—H2A	0.9900	C29—C30	1.392 (2)
C2—H2B	0.9900	C30—H30	0.9500
C3—H3A	0.9900	C30—C31	1.386 (2)
C3—H3B	0.9900	C31—H31	0.9500
C3—C4	1.504 (3)	C31—C32	1.378 (2)
C4—C5	1.386 (3)	C32—H32	0.9500
C5—H5	0.9500	C32—C33	1.389 (2)
C5—C6	1.372 (3)	C33—H33	0.9500
C6—H6	0.9500	C34—C35	1.404 (2)
C6—C7	1.379 (4)	C34—C39	1.406 (2)
C7—H7	0.9500	C34—B1	1.644 (2)
C7—C8	1.377 (3)	C35—H35	0.9500
C8—H8	0.9500	C35—C36	1.391 (2)
C9—H9	0.9500	C36—H36	0.9500
C9—C10	1.378 (3)	C36—C37	1.379 (3)
C10—H10	0.9500	C37—H37	0.9500
C10—C11	1.379 (2)	C37—C38	1.387 (2)
C11—H11	0.9500	C38—H38	0.9500
C11—C12	1.375 (2)	C38—C39	1.385 (2)
C12—H12	0.9500	C39—H39	0.9500
C13—H13A	0.9900	C40—C41	1.389 (2)
C13—H13B	0.9900	C40—C45	1.403 (2)
C13—C14	1.505 (2)	C40—B1	1.643 (2)
C14—C15	1.386 (2)	C41—H41	0.9500
C15—H15	0.9500	C41—C42	1.398 (2)
C15—C16	1.382 (3)	C42—H42	0.9500
C16—H16	0.9500	C42—C43	1.372 (3)
C16—C17	1.379 (3)	C43—H43	0.9500
C17—H17	0.9500	C43—C44	1.378 (3)
C17—C18	1.374 (2)	C44—H44	0.9500
C18—H18	0.9500	C44—C45	1.391 (3)
C19—C20	1.502 (2)	C45—H45	0.9500
			
O1—Mn1—O2	81.52 (4)	C18—C17—C16	118.46 (16)
O1—Mn1—O3	101.03 (5)	C18—C17—H17	120.8
O1—Mn1—N1	88.33 (5)	N4—C18—C17	122.91 (15)
O1—Mn1—N2	92.28 (5)	N4—C18—H18	118.5
O1—Mn1—N3	88.03 (4)	C17—C18—H18	118.5
O1—Mn1—N4	166.95 (5)	O2—C19—O3	120.79 (15)
O2—Mn1—O3	54.74 (4)	O2—C19—C20	120.41 (15)
O3—Mn1—N1	132.87 (5)	O3—C19—C20	118.79 (16)
O3—Mn1—N2	152.00 (5)	C19—C20—H20A	109.5
O3—Mn1—N3	83.91 (5)	C19—C20—H20B	109.5
O3—Mn1—N4	88.91 (5)	C19—C20—H20C	109.5
N1—Mn1—O2	81.88 (5)	H20A—C20—H20B	109.5
N1—Mn1—N2	71.41 (5)	H20A—C20—H20C	109.5
N1—Mn1—N3	142.97 (5)	H20B—C20—H20C	109.5
N2—Mn1—O2	152.77 (5)	O1—C21—H21A	109.5
N3—Mn1—O2	133.76 (4)	O1—C21—H21B	109.5
N3—Mn1—N2	71.94 (5)	O1—C21—H21C	109.5
N4—Mn1—O2	111.30 (4)	H21A—C21—H21B	109.5
N4—Mn1—N1	91.07 (5)	H21A—C21—H21C	109.5
N2—Mn1—N4	75.20 (4)	H21B—C21—H21C	109.5
N4—Mn1—N3	84.61 (4)	C23—C22—C27	115.29 (15)
Mn1—O1—H1	115.2 (11)	C23—C22—B1	124.68 (15)
C21—O1—Mn1	129.43 (12)	C27—C22—B1	120.02 (13)
C21—O1—H1	106.3 (11)	C22—C23—H23	118.9
C19—O2—Mn1	85.21 (9)	C24—C23—C22	122.17 (19)
C19—O3—Mn1	99.20 (11)	C24—C23—H23	118.9
C4—N1—Mn1	117.72 (11)	C23—C24—H24	119.9
C8—N1—Mn1	123.44 (13)	C25—C24—C23	120.28 (19)
C8—N1—C4	118.83 (16)	C25—C24—H24	119.9
C2—N2—Mn1	109.13 (9)	C24—C25—H25	120.2
C2—N2—C13	112.02 (13)	C24—C25—C26	119.65 (17)
C3—N2—Mn1	106.06 (10)	C26—C25—H25	120.2
C3—N2—C2	110.69 (13)	C25—C26—H26	120.2
C3—N2—C13	110.45 (13)	C25—C26—C27	119.6 (2)
C13—N2—Mn1	108.28 (9)	C27—C26—H26	120.2
C1—N3—Mn1	118.77 (10)	C22—C27—H27	118.5
C12—N3—Mn1	122.76 (10)	C26—C27—C22	122.97 (17)
C12—N3—C1	118.23 (13)	C26—C27—H27	118.5
C14—N4—Mn1	117.29 (10)	C29—C28—C33	114.96 (13)
C18—N4—Mn1	123.01 (10)	C29—C28—B1	124.89 (13)
C18—N4—C14	118.46 (13)	C33—C28—B1	120.14 (13)
N3—C1—C2	116.93 (14)	C28—C29—H29	118.6
N3—C1—C9	122.07 (15)	C30—C29—C28	122.70 (14)
C9—C1—C2	120.91 (14)	C30—C29—H29	118.6
N2—C2—C1	112.94 (13)	C29—C30—H30	119.7
N2—C2—H2A	109.0	C31—C30—C29	120.52 (15)
N2—C2—H2B	109.0	C31—C30—H30	119.7
C1—C2—H2A	109.0	C30—C31—H31	120.8
C1—C2—H2B	109.0	C32—C31—C30	118.45 (14)
H2A—C2—H2B	107.8	C32—C31—H31	120.8
N2—C3—H3A	109.4	C31—C32—H32	119.8
N2—C3—H3B	109.4	C31—C32—C33	120.48 (15)
N2—C3—C4	111.33 (14)	C33—C32—H32	119.8
H3A—C3—H3B	108.0	C28—C33—H33	118.6
C4—C3—H3A	109.4	C32—C33—C28	122.87 (15)
C4—C3—H3B	109.4	C32—C33—H33	118.6
N1—C4—C3	116.60 (15)	C35—C34—C39	114.69 (14)
N1—C4—C5	121.68 (18)	C35—C34—B1	124.20 (13)
C5—C4—C3	121.66 (18)	C39—C34—B1	121.01 (13)
C4—C5—H5	120.4	C34—C35—H35	118.7
C6—C5—C4	119.1 (2)	C36—C35—C34	122.57 (15)
C6—C5—H5	120.4	C36—C35—H35	118.7
C5—C6—H6	120.4	C35—C36—H36	119.7
C5—C6—C7	119.2 (2)	C37—C36—C35	120.60 (16)
C7—C6—H6	120.4	C37—C36—H36	119.7
C6—C7—H7	120.5	C36—C37—H37	120.6
C8—C7—C6	119.1 (2)	C36—C37—C38	118.89 (15)
C8—C7—H7	120.5	C38—C37—H37	120.6
N1—C8—C7	122.1 (2)	C37—C38—H38	120.1
N1—C8—H8	119.0	C39—C38—C37	119.87 (15)
C7—C8—H8	119.0	C39—C38—H38	120.1
C1—C9—H9	120.5	C34—C39—H39	118.3
C10—C9—C1	118.90 (15)	C38—C39—C34	123.35 (14)
C10—C9—H9	120.5	C38—C39—H39	118.3
C9—C10—H10	120.4	C41—C40—C45	114.91 (15)
C9—C10—C11	119.28 (15)	C41—C40—B1	124.17 (14)
C11—C10—H10	120.4	C45—C40—B1	120.91 (14)
C10—C11—H11	120.8	C40—C41—H41	118.5
C12—C11—C10	118.34 (16)	C40—C41—C42	123.04 (16)
C12—C11—H11	120.8	C42—C41—H41	118.5
N3—C12—C11	123.17 (15)	C41—C42—H42	120.0
N3—C12—H12	118.4	C43—C42—C41	120.06 (18)
C11—C12—H12	118.4	C43—C42—H42	120.0
N2—C13—H13A	108.4	C42—C43—H43	120.5
N2—C13—H13B	108.4	C42—C43—C44	119.05 (17)
N2—C13—C14	115.31 (13)	C44—C43—H43	120.5
H13A—C13—H13B	107.5	C43—C44—H44	119.9
C14—C13—H13A	108.4	C43—C44—C45	120.23 (18)
C14—C13—H13B	108.4	C45—C44—H44	119.9
N4—C14—C13	118.13 (13)	C40—C45—H45	118.6
N4—C14—C15	121.82 (15)	C44—C45—C40	122.71 (18)
C15—C14—C13	119.89 (14)	C44—C45—H45	118.6
C14—C15—H15	120.6	C22—B1—C28	110.31 (12)
C16—C15—C14	118.85 (16)	C34—B1—C22	108.42 (12)
C16—C15—H15	120.6	C34—B1—C28	110.47 (11)
C15—C16—H16	120.3	C40—B1—C22	110.69 (12)
C17—C16—C15	119.44 (16)	C40—B1—C28	107.22 (12)
C17—C16—H16	120.3	C40—B1—C34	109.72 (12)
C16—C17—H17	120.8		
			
Mn1—O2—C19—O3	−2.16 (14)	C23—C22—B1—C28	−109.03 (17)
Mn1—O2—C19—C20	178.52 (15)	C23—C22—B1—C34	129.87 (15)
Mn1—O3—C19—O2	2.48 (16)	C23—C22—B1—C40	9.5 (2)
Mn1—O3—C19—C20	−178.19 (13)	C23—C24—C25—C26	1.4 (3)
Mn1—N1—C4—C3	−0.32 (19)	C24—C25—C26—C27	−0.4 (3)
Mn1—N1—C4—C5	−177.45 (13)	C25—C26—C27—C22	−1.7 (3)
Mn1—N1—C8—C7	177.02 (14)	C27—C22—C23—C24	−1.6 (2)
Mn1—N2—C2—C1	35.27 (16)	C27—C22—B1—C28	70.36 (17)
Mn1—N2—C3—C4	−43.91 (15)	C27—C22—B1—C34	−50.73 (17)
Mn1—N2—C13—C14	−22.55 (17)	C27—C22—B1—C40	−171.13 (14)
Mn1—N3—C1—C2	8.77 (17)	C28—C29—C30—C31	−0.4 (2)
Mn1—N3—C1—C9	−174.82 (11)	C29—C28—C33—C32	0.1 (2)
Mn1—N3—C12—C11	174.96 (12)	C29—C28—B1—C22	−15.7 (2)
Mn1—N4—C14—C13	−18.61 (18)	C29—C28—B1—C34	104.11 (16)
Mn1—N4—C14—C15	166.15 (12)	C29—C28—B1—C40	−136.36 (14)
Mn1—N4—C18—C17	−167.80 (13)	C29—C30—C31—C32	0.3 (2)
N1—C4—C5—C6	0.3 (3)	C30—C31—C32—C33	0.1 (3)
N2—C3—C4—N1	32.0 (2)	C31—C32—C33—C28	−0.2 (3)
N2—C3—C4—C5	−150.90 (16)	C33—C28—C29—C30	0.3 (2)
N2—C13—C14—N4	28.7 (2)	C33—C28—B1—C22	163.65 (13)
N2—C13—C14—C15	−155.92 (15)	C33—C28—B1—C34	−76.48 (17)
N3—C1—C2—N2	−30.8 (2)	C33—C28—B1—C40	43.04 (18)
N3—C1—C9—C10	0.0 (2)	C34—C35—C36—C37	0.6 (3)
N4—C14—C15—C16	2.5 (2)	C35—C34—C39—C38	1.4 (2)
C1—N3—C12—C11	0.6 (2)	C35—C34—B1—C22	146.46 (14)
C1—C9—C10—C11	−0.1 (2)	C35—C34—B1—C28	25.47 (19)
C2—N2—C3—C4	−162.15 (14)	C35—C34—B1—C40	−92.54 (16)
C2—N2—C13—C14	97.84 (16)	C35—C36—C37—C38	0.9 (3)
C2—C1—C9—C10	176.25 (15)	C36—C37—C38—C39	−1.2 (2)
C3—N2—C2—C1	151.62 (14)	C37—C38—C39—C34	0.0 (2)
C3—N2—C13—C14	−138.27 (15)	C39—C34—C35—C36	−1.7 (2)
C3—C4—C5—C6	−176.65 (17)	C39—C34—B1—C22	−37.39 (18)
C4—N1—C8—C7	−1.7 (3)	C39—C34—B1—C28	−158.39 (13)
C4—C5—C6—C7	−1.7 (3)	C39—C34—B1—C40	83.61 (16)
C5—C6—C7—C8	1.3 (3)	C40—C41—C42—C43	−0.6 (3)
C6—C7—C8—N1	0.4 (3)	C41—C40—C45—C44	−0.1 (3)
C8—N1—C4—C3	178.47 (15)	C41—C40—B1—C22	107.07 (16)
C8—N1—C4—C5	1.3 (2)	C41—C40—B1—C28	−132.55 (14)
C9—C1—C2—N2	152.78 (14)	C41—C40—B1—C34	−12.55 (19)
C9—C10—C11—C12	0.4 (2)	C41—C42—C43—C44	0.6 (3)
C10—C11—C12—N3	−0.7 (2)	C42—C43—C44—C45	−0.4 (3)
C12—N3—C1—C2	−176.64 (13)	C43—C44—C45—C40	0.1 (3)
C12—N3—C1—C9	−0.2 (2)	C45—C40—C41—C42	0.3 (2)
C13—N2—C2—C1	−84.62 (16)	C45—C40—B1—C22	−72.30 (18)
C13—N2—C3—C4	73.20 (17)	C45—C40—B1—C28	48.07 (18)
C13—C14—C15—C16	−172.67 (16)	C45—C40—B1—C34	168.08 (14)
C14—N4—C18—C17	−0.9 (2)	B1—C22—C23—C24	177.84 (16)
C14—C15—C16—C17	−1.1 (3)	B1—C22—C27—C26	−176.78 (15)
C15—C16—C17—C18	−1.1 (3)	B1—C28—C29—C30	179.69 (14)
C16—C17—C18—N4	2.2 (3)	B1—C28—C33—C32	−179.38 (14)
C18—N4—C14—C13	173.78 (14)	B1—C34—C35—C36	174.68 (15)
C18—N4—C14—C15	−1.5 (2)	B1—C34—C39—C38	−175.12 (14)
C22—C23—C24—C25	−0.4 (3)	B1—C40—C41—C42	−179.08 (14)
C23—C22—C27—C26	2.7 (2)	B1—C40—C45—C44	179.36 (17)

### Hydrogen-bond geometry (Å, º)

Cg1, Cg2, Cg3, Cg4, Cg6, and Cg7 are the centroids of the N1/C4–C8, N3/C1/C9/C10–C12, N4/C14–C18, C22–C27, C34–C39, and C40–C45 rings, respectively.

**Table:**

*D*—H···*A*	*D*—H	H···*A*	*D*···*A*	*D*—H···*A*
O1—H1···O2^i^	0.86 (1)	1.79 (1)	2.6480 (17)	176 (2)
C8—H8···O2	0.95	2.45	3.056 (2)	121
C12—H12···O3	0.95	2.35	2.987 (2)	124
C2—H2*B*···*Cg*6^ii^	0.99	2.70	3.6260 (18)	156
C38—H38···*Cg*4^iii^	0.95	2.81	3.7135 (19)	158
C42—H42···*Cg*3^iv^	0.95	2.96	3.659 (2)	131
*Cg*1···*Cg*7^iv^			4.2073 (11)	
*Cg*2···*Cg*3			4.6125 (10)	
*Cg*3···*Cg*4^v^			4.2267 (12)	
*Cg*4···*Cg*6^iii^			5.0645 (11)	

Symmetry codes: (i) −*x*+1, −*y*, −*z*+1; (ii) *x*, *y*, *z*−1; (iii) −*x*+2, −*y*+1, −*z*+2; (iv) −*x*+2, −*y*+1, −*z*+1; (v) −*x*+1, −*y*+1, −*z*+1.

### Selected bond lengths (Å) and angles (°) of the title compound

**Table:**

Mn1—O1	2.1941 (12)	Mn1—N4	2.2496 (13)
Mn1—O2	2.5009 (12)	O1—Mn1—N4	166.95 (5)
Mn1—O3	2.2004 (13)	O2—Mn1—O3	54.74 (4)
Mn1—N1	2.2769 (15)	N2—Mn1—N4	75.20 (4)
Mn1—N2	2.4092 (13)	O1—Mn1—O2	81.52 (4)
Mn1—N3	2.3022 (13)		
